# Pain assessment during blood collection from sedated and mechanically ventilated children

**DOI:** 10.5935/0103-507X.20160013

**Published:** 2016

**Authors:** Layra Viviane Rodrigues Pinto Dantas, Thiago Silveira Pinto Dantas, Valter Joviniano Santana-Filho, Isabela Freire Azevedo-Santos, Josimari Melo DeSantana

**Affiliations:** 1 Postgraduate Program in Health Sciences, Universidade Federal de Sergipe - Aracaju (SE), Brazil.; 2 Faculdade Estácio de Sergipe - Aracaju (SE), Brazil.; 3 Department of Physical Therapy, Universidade Federal de Sergipe - Aracaju (SE), Brazil.

**Keywords:** Pain measurement, Respiration, artificial, Intensive care units, pediatric, Child, Child, preschool

## Abstract

**Objective:**

This study assessed pain and observed physiological parameters in sedated and mechanically ventilated children during a routine procedure.

**Methods:**

This observational study was performed in a pediatric intensive care unit. Thirty-five children between 1 month and 12 years of age were assessed before, during, and five minutes after an arterial blood collection for gas analysis (painful procedure). Face, Legs, Activity, Cry and Consolability scale was used to assess pain. In addition, patients' heart rate, respiratory rate, peripheral saturation of oxygen and blood pressure (diastolic and systolic) were recorded. COMFORT-B scale was applied before the pain and physiological parameter assessments to verify sedation level of the subjects.

**Results:**

There was an increase in Face, Legs, Activity, Cry and Consolability score (p = 0.0001) during painful stimuli. There was an increase in heart rate (p = 0.03), respiratory rate (p = 0.001) and diastolic blood pressure (p = 0.006) due to pain caused by the routine procedure.

**Conclusions:**

This study suggests that assessments of pain using standard scales, such as Face, Legs, Activity, Cry and Consolability score, and other physiological parameters should be consistently executed to optimize pain management in pediatric intensive care units.

## Abstract (pt)

**Objetivo:**

Avaliar a dor e observar parâmetros fisiológicos em crianças sedadas e submetidas à ventilação mecânica durante um procedimento de rotina.

**Métodos:**

Estudo observacional realizado em uma unidade de terapia intensiva pediátrica. Foram avaliadas 35 crianças, com idades entre 1 mês e 12 anos, em três momentos distintos: antes, durante e 5 minutos após coleta de sangue arterial para análise gasométrica (procedimento doloroso). Utilizou-se a Escala *Face, Legs, Activity, Cry and Consolability* para avaliação da dor e foram registradas a frequência cardíaca, frequência respiratória, saturação periférica de oxigênio e pressão arterial (sistólica e diastólica). O nível de sedação dos participantes foi verificado utilizando-se a escala Comfort-B, aplicada antes da mensuração da dor e da avaliação dos parâmetros fisiológicos.

**Resultados:**

Durante os estímulos dolorosos, ocorreu aumento do escore da Escala *Face, Legs, Activity, Cry and Consolability* (p = 0,0001). Houve também aumento da frequência cardíaca (p = 0,03), da frequência respiratória (p = 0,001) e da pressão arterial diastólica (p = 0,006) em razão da dor causada pelo procedimento de rotina.

**Conclusões:**

Avaliação da dor com uso de escalas padrão, como a Escala *Face, Legs, Activity, Cry and Consolability*, e observação de parâmetros fisiológicos, deve ser realizada rotineiramente para melhorar o manejo da dor nas unidades de terapia intensiva pediátricas.

## INTRODUCTION

The association between pain and negative physiological, emotional and psychological symptoms is well established.^(1)^ The myths that children do not feel pain in a similar manner to adults and that pain does not cause problematic consequences prevail,^(2)^ despite the evidence of pain perception beginning at the gestational period.^(3)^

For an adequate pain management, an appropriate assessment is necessary. Pain in children is underestimated due to a lack of adequate assessment tools based on different developmental stages and clinical conditions and the fear of oversedation, respiratory depression, addiction or unfamiliarity with use of sedative and analgesic agents in children.^(4)^

In pediatric intensive care units (PICU), pain assessment is difficult, particularly in sedated patients under mechanical ventilation. It is often not possible to distinguish between pain and anxiety, even though both must be treated at the same time. Mechanically ventilated newborns and children are exposed to acute illnesses. The PICU environment can produce anxiety and pain caused by routine procedures, such as tracheal suctioning, arterial blood collection and venipuncture.^(5)^ However, sedoanalgesia is not an adequate treatment^(6)^ because of the difficulty in measuring pain with the scales developed and validated for this population. In addition, a lack of clinical knowledge, insufficient studies and unknown side effects caused by opioids make effective pain control an unusual practice in the PICU.

Face, Legs, Activity, Cry and Consolability (FLACC) behavioral scale was developed to reduce the existing barriers in the measurement of pain in children. This scale is considered easy to apply and shows excellent validity and reliability when used to demonstrate a change in pain scores before and after analgesic medicine administration in children.^(7-12)^

This scale was previously used in sedated and mechanically ventilated children on two occasions: Modified FLACC and COMFORT-B scales were used to analyze psychometrical properties in the Swedish population,^(11)^ and pain was measured by nurses during endotracheal suction.^(12)^ However, no published studies have used the original FLACC scale to assess pain in children unable to self-report during other routine PICU procedures. Thus, this study assessed pain and measured physiological parameters in pediatric patients sedated and mechanically ventilated during a routine PICU procedure (arterial blood collection for gas analysis).

## METHODS

This descriptive and observational study was performed in the PICU at the *Hospital de Urgências de Sergipe* in Aracaju, Sergipe, Brazil. After approval by the *Universidade Federal de Sergipe* Ethics Committee (CAAE: 6139.0.000.107-10), patients' legal conservators were informed about the aims of the study, and signed consent forms were obtained for all participants.

Participants were chosen from patients admitted to the PICU based on inclusion/exclusion criteria. Children under sedation and mechanical ventilation (assisted-control mode) between 1 month and 12 years of age were included. Victims of trauma with a level lower than 8 on Glasgow coma scale and patients with drug-induced neuromuscular blockade were excluded. All subjects were recruited prior to an arterial blood collection for gas analysis. This invasive sample collection technique is routinely performed to monitor mechanically ventilated patients. The blood draw was performed using a 1mL syringe with a subcutaneous needle and no local anesthetics. The amount of blood collected varied between 0.5 and 1mL.

The COMFORT-B scale^(13)^ was used to verify the sedation level. Based on this scale, patients can be classified as "excessively sedated" (score between 6 and 10), "sedated" (scores between 11 and 22) and "insufficiently sedated" (scores higher than 23). In this study, only patients considered "sedated" were included.

After establishing the sedation level, pain and physiological parameters were assessed by a single investigator at three different time points: immediately before the arterial blood collection for gas analysis (TPre), during the routine procedure (T0) and five minutes after the procedure (T5).

The FLACC scale^(10)^ was used to measure pain. Each of the five categories used in this assessment is scored from 0 to 2 with a total score ranging from 0 to 10. A score of "0" is considered as relaxed or comfortable, "1 - 3" represents mild discomfort, "4 - 6" represents moderate pain and "7 - 10" signifies severe pain and/or discomfort. The cardiac monitor Dash 4000 was used to monitor the respiratory rate (RR), heart rate (HR), peripheral oxygen saturation (SpO_2_) and blood pressure (BP) of the children.

### Statistical analysis

The software Statistical Package for the Social Science (SPSS) version 19.0 was used for statistical analysis. The Shapiro-Wilk test was used to verify data normality. Quantitative data are presented as the mean and standard deviation (parametric) or as the median and 25^th^ and 75^th^ percentile (non-parametric). Categorical data are represented as the frequency or total count.

To compare FLACC scores from the three assessment moments, the Friedman's test followed by the Dunn's post-hoc test was used. Comparisons between pain intensity and physiological parameters were performed using a repeated-measure ANOVA adjusted for age and Bonferroni post-hoc tests adjusted for confidence intervals (95%CI).

Cronbach's alpha was calculated to analyze intra-rater reliability across the three assessments completed by a single investigator. A p-value ≤ 0.05 was considered statistically significant, the power was set at 0.80, and all tests were two-tailed.

## RESULTS

Thirty-five patients with a mean age of 5 months (p25 = 3 months and p75 = 19 months) were assessed. The frequency of males and females was 60% and 40%, respectively. The main diagnoses included seven patients (20%) with pneumonia, 6 patients (17.1%) with acute respiratory insufficiency, 5 patients (14.3%) with sepsis and 4 patients (11.4%) with congenital heart disease.

Midazolam was the most frequently used drug for sedoanalgesia (77.1%), followed by fentanyl (65.7%), phenytoin and phenobarbital (14.3% each). All study participants were considered sedated (n = 35) with a COMFORT-B score between 11 and 22 (median = 13, p25 = 12 and p75 = 15). None of the subjects were excessively or insufficiently sedated during the assessment. Only 3 (8.6%) children received analgesic medication prior to the painful procedure.

**Figure 1:**
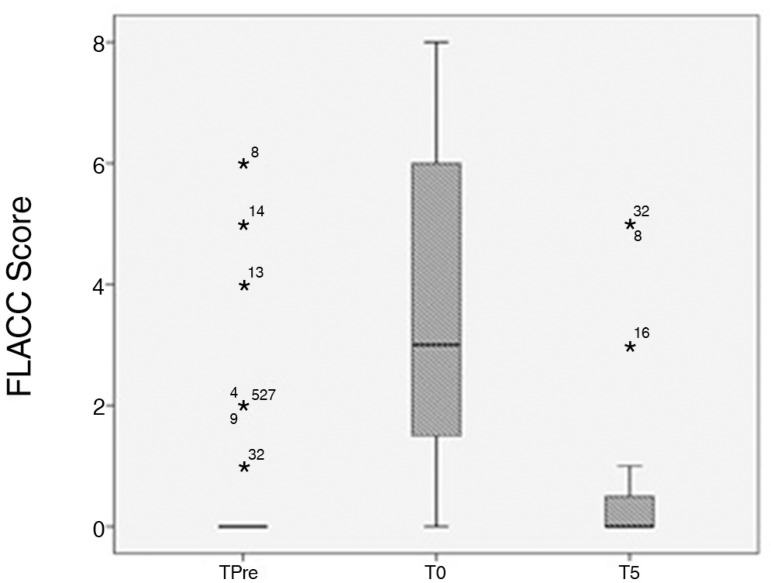
Face, Legs, Activity, Cry and Consolability scores from three assessments: before the painful procedure, during the painful procedure, and 5 minutes after the painful procedure. The values are shown as the median and percentile. FLACC - Face, Legs, Activity, Cry and Consolability scale; TPre - before the painful procedure, T0 - during the painful procedure; T5 - 5 minutes after the painful procedure. * p < 0.05 between T0-TPre and T0-T5 (Friedman’s test followed by Dunn’s post-hoc test).

The FLACC scores varied from 0 to 6 with means of 0 (TPre), 3 (T0) and 0 (T5). During blood collection (T0), the pain intensity was significantly higher than before the procedure (TPre moment) and five minutes after the procedure (T5) (p = 0.0001). However, no difference was observed between the pain intensities recorded at TPre and T5 (Figure 1 and Table 1).

**Table 1:** Face, Legs, Activity, Cry and Consolability scores and physiological parameters from three assessments

**Variables**	**TPre**	**T0**	**T5**	**p value**
FLACC*	0 (0; 0)	3 (1; 6)	0 (0; 1)	0.0001
HR^†^	133.9 ± 28.6	143.1 ± 28.5	134.8 ± 26.1	0.03
RR^†^	34.0 ± 10.0	37 ± 10.0	33 ± 8.0	0.001
SpO_2_^†^	96.7 ± 3.5	96.2 ± 3.7	97.2 ± 3.0	0.4
SBP^†^	97.6 ± 20.2	102.4 ± 27.8	98.2 ± 24.9	0.45
DBP^†^	56.3 ± 15.8	63.4 ± 19.4	61 ± 16.8	0.006

TPre - prior to the painful procedure; T0 - during the painful procedure; T5 - five minutes after the painful procedure; FLACC - Face, Legs, Activity, Cry and Consolability scale; HR - heart rate; RR - respiratory rate; SpO_2_ - peripheral oxygen saturation; SBP - systolic blood pressure; DBP - diastolic blood pressure. Data are represented as the mean, p25 and p75. ;

* Friedman test

† repeated-measures ANOVA.

**Figure 2:**
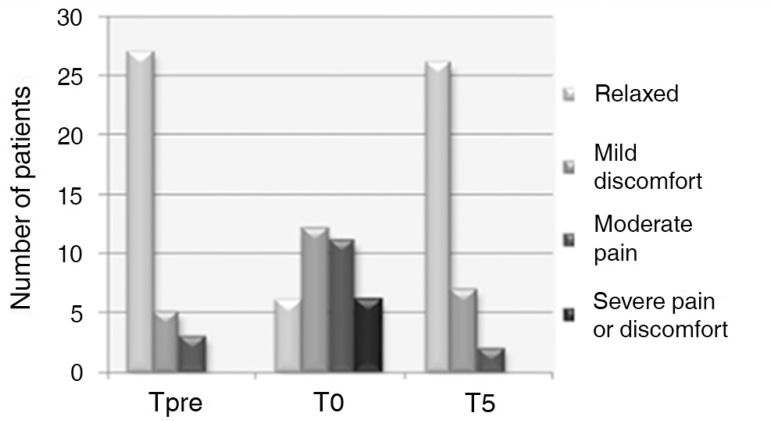
Sample distribution according to the Face, Legs, Activity, Cry and Consolability categories. TPre - before the painful procedure; T0 - during the painful procedure; T5 - five minutes after the painful procedure.

Approximately 83% of the patients presented with a painful perception during blood collection (T0) that varied from a small level of discomfort to an intense pain (Figure 2). An intra-rater reliability analysis for a single investigator between time points resulted in a Cronbach's alpha value of 0.706.

With regards to the assessed physiological parameters, HR was significantly higher immediately after the painful stimulus (T0) when compared with the other time points (p = 0.03; Table 1). Similarly, the mean respiratory rate was also significantly higher after the painful stimulus (T0) when compared with the other time points (p = 0.0001; Table 1). There was no difference in the SpO_2_ levels between time points (p = 0.4) (Table 1).

The systolic blood pressure (SBP) showed a homogenous distribution across the three assessments (p = 0.45). The diastolic blood pressure (DBP) was significantly higher at T0 (p = 0.006). The DBP at T5 what higher than at TPre (4.7 ± 2.1); however, the difference was not statistically significant (p = 0.09).

## DISCUSSION

Pain assessment in sedated and mechanically ventilated pediatric patients using validated tools is considered important for clinical practice. The necessity for a specific pain scale in the PICU was evidenced in the present study. In our study, the FLACC scale was chosen because of its large applicability to a wide range of ages within the pediatric population. In accordance with previous studies, the FLACC scale is effective as a pain assessment tool for use by hospital units (e.g., oncology and trauma) in children less than 3 years old.^(14)^ In addition, this tool is valid and reliable for the assessment of postoperative pain assessment in children and teenagers (from 4 to 18 years old) with cognitive impairment.^(15)^

Our results showed a significant variation in FLACC scores during an arterial blood collection for gas analysis from mechanically ventilated and sedated children. These data suggest that despite the use of sedoanalgesia and an adequate COMFORT-B sedation score, patients exhibited a FLACC score ranging from mild discomfort to moderate pain. Similarly, Babl et al.^(16)^ studied the importance of nebulization with lidocaine for reducing pain intensity during nasogastric tube insertion in children. This group observed that the FLACC scores were higher during this procedure and that the lidocaine group (local anesthesia) had lower scores when compared with the control group.

In addition to intubation, other painful procedures are performed in the PICU without analgesia. These methods have been reported in previous studies^(4,17-21)^ and are routinely observed in intensive care units, especially in patients under sedation, due to the completely erroneous impression that the patient does not feel pain. Indeed, we clearly observed that behavioral and physiological parameters changed when a painful stimulus was applied to our subjects.

Based on our findings, the FLACC scale can be used to measure pain in sedated and mechanically ventilated children with high reliability (Cronbach's alpha = 0.706). These findings are in accordance with previous FLACC assessments collected from children and adolescents in Brazil (Cronbach alfa = 0.76).^(10)^ Similarly, Voepel-Lewis^(22)^ tested the reliability and validity of this scale in critically ill adults and children unable to self-report pain (intubated or with cognitive impairment) and reported a Cronbach's alpha of 0.882. Darnell et al.^(23)^ and Babl et al.^(16)^ reported that the FLACC scale is a valid and reliable assessment tool for pain in the pediatric population and pain related to procedures in children at pre-verbal and verbal ages.

In conjunction with the variation in FLACC scores, we observed an abrupt increase in RR and HR during the painful procedure. Aïssaoui et al.^(24)^ also observed a significant increase in HR (10%) and BP during painful procedures in patients sedated and mechanically ventilated. Additionally, Weissman et al.^(25)^ reported that HR is frequently used as a physiological parameter for harmful events and as a complementary measure to detect autonomous nervous system conditions during painful procedures. In contrast, Pereira et al.^(26)^ observed a decrease in HR during and one minute after a painful procedure (venipuncture and alcohol rubbing on the dorsum of the hand) in newborns in intensive care units. Notably, a subsequent increase in HR at 5 minutes after the procedure was observed by Pereira et al. Our study indicated that the HR returned to baseline by this time (five minutes after the painful procedure).

Changes in peripheral oxygen saturation (SpO_2_) levels were not found in this study, and thus cannot be considered as a valid parameter for the direct assessment of pain in mechanically ventilated children. In contrast, two previous studies^(12,26)^ observed a consistent and acute decrease in oxygen saturation levels during^(12)^ and right after^(26)^ a painful procedure. These data suggest that saturation levels are a valid parameter for pain assessment in newborns and children. However, these studies also highlight that SpO_2_ levels may have a low specificity because it can be changed by other non-painful causes in these subjects.

In our study, the SBP was maintained with no significant changes. Unlike other physiological parameters, the DBP increased during the painful stimulus and remained high five minutes after the procedure. Jeitziner et al.^(27)^ observed an increase in SBP during a painful procedure (tracheal aspiration), even when it was performed with the application of analgesics in critically ill subjects. In addition, the DBP increased during the painful procedure, but this increase could be counteracted with the application of analgesics.^(27)^ Miranda et al.^(28)^ demonstrated similar results in postoperative cardiac patients. However, unlike our study, this group found no significant correlation between SBP, DBP and pain.

Acute pain has a biological purpose, including signaling that an organic injury has occurred and stimulating the sympathetic nervous system to evoke physiological responses, such as an increase in HR, BP and RR.^(12,29)^ Thus, we expected an increase in the SBP and DBP of our patients. Partly confirming our findings, Buttner and Finke^(29)^ affirmed that physiological parameters, such as HR, RR and blood pressure, have little discriminating power to the detect postoperative analgesia need in newborns, infants and young children.

Johansson and Kokinsky^(11)^ proved inter-observer reliability and the construct validity of the Swedish COMFORT-B and modified FLACC scales (with changes in "cry" item) by detected a decrease in pain intensity after morphine administration in postoperative children. Furthermore, Sönmez and Kuğuoğlu^(12)^ demonstrated the importance of pain control and sedation by detecting lower FLACC and Wong-Baker Faces scores in subjects who received analgesia and a bolus of sedatives during endotracheal suctioning when compared with patients who did not receive pain management. In accordance with our findings, these studies show that pain and sedation assessments can be used in conjunction to improve pain management in the PICU.

## CONCLUSION

We conclude that mechanically ventilated and sedated children feel pain, as demonstrated by the Face, Legs, Activity, Cry and Consolability scale. Our study shows that this scale is reliable for measuring pain in children who are intubated and unable to self-report. High pain intensity scores and changes in heart rate, respiratory rate and diastolic blood pressure during an arterial blood collection for gas analysis confirm our hypothesis. Thus, pain should be assessed in a multidimensional approach by incorporating physiological parameters, which separately are nonspecific, with objective measurements based on standardized scales that provide information on individual pain responses.

Healthcare professionals often assume that sedation is an adequate routine practice that provides effective pain control; however, a child´s inability to verbally express discomfort does not mean that pain is not being experienced. Thus, this study aims to alert health professionals to the importance of appropriately assessing pain in children, especially in patients for whom it is difficult to verbally expressing painful sensations.
